# Neural Networks in Autosomal Dominant Alzheimer’s Disease: Insights From Functional Magnetic Resonance Imaging Studies

**DOI:** 10.3389/fnagi.2022.903269

**Published:** 2022-07-19

**Authors:** Qiongqiong Qiu

**Affiliations:** ^1^Innovation Center for Neurological Disorders, Department of Neurology, Xuanwu Hospital, Capital Medical University, Beijing, China; ^2^Beijing Key Laboratory of Geriatric Cognitive Disorders, Beijing, China; ^3^Clinical Center for Neurodegenerative Disease and Memory Impairment, Capital Medical University, Beijing, China; ^4^Center of Alzheimer’s Disease, Beijing Institute for Brain Disorders, Beijing, China; ^5^Key Laboratory of Neurodegenerative Diseases, Ministry of Education, National Clinical Research Center for Geriatric Disorders, Beijing, China

**Keywords:** autosomal dominant Alzheimer’s disease, neural network, functional MRI (fMRI), presenilin, amyloid precursor protein (APP)

## Abstract

Alzheimer’s disease (AD) is the most common form of dementia, with no cure to stop its progression. Early detection, diagnosis, and intervention have become the hot spots in AD research. The long asymptomatic and slightly symptomatic phases of autosomal dominant AD (ADAD) allow studies to explore early biomarkers and the underlying pathophysiological changes. Functional magnetic resonance imaging (fMRI) provides a method to detect abnormal patterns of brain activity and functional connectivity *in vivo*, which correlates with cognitive decline earlier than structural changes and more strongly than amyloid deposition. Here, we will provide a brief overview of the network-level findings in ADAD in fMRI studies. In general, abnormalities in brain activity were mainly found in the hippocampus, the medial temporal lobe (MTL), the posterior cortex, the cingulate cortices, and the frontal regions in ADAD. Moreover, ADAD and sporadic AD (SAD) have similar fMRI changes, but not with aging.

## Introduction

Alzheimer’s disease (AD) is a neurodegenerative disorder characterized by the progressive loss of cognitive function and independence, with extracellular plaque deposits of the β-amyloid peptide (Aβ) and flame-shaped neurofibrillary tangles of the microtubule-binding protein tau. Autosomal dominant AD (ADAD) accounts for less than 1% of all AD cases (Bekris et al., 2010), which is attributed to mutations in three genes: amyloid precursor protein (*APP*), presenilin 1 (*PSEN1*), and presenilin 2 (*PSEN2*). Mutation carriers usually develop dementia at an early age (about 30 to 50 years of age), while sporadic AD (SAD) subjects develop symptoms of progressive amnesia and other cortical cognitive symptoms, usually after age 60. With mutation-specific exceptions and an earlier age of onset, ADAD is similar to SAD. Owing to the nearly 100% penetrance of ADAD, it is possible to identify early biomarkers and explore the initial pathophysiological mechanisms in cognitively unimpaired ADAD mutation carriers. Furthermore, studies that recruit subjects in pre-senile, mild-adulthood, or even childhood can reduce the impact of aging and other comorbidities on the results of functional magnetic resonance imaging (fMRI) research.

Functional magnetic resonance imaging is a technique for measuring and mapping brain activity and functional connectivity. It can be conducted in the resting state (rs-fMRI) or during the performance of particular cognitive tasks (task-related fMRI), which allows the assessment of intrinsic activity and polysynaptic connections in the brain. Task-based and resting-state represent the two most common experimental paradigms of functional neuroimaging. Task-related fMRI is model-driven and rs-fMRI analysis is data-driven. They can reflect different features of functional changes in the brain. By comparing the differences in the activation areas of different groups on the task, topics such as disease, development, and cognition can be analyzed. The rs-fMRI analysis is data-driven, and its analytical approach focuses on describing the synergistic and spontaneous activity of the brain. Blood oxygenation level-dependent (BOLD) imaging is the standard technique used to generate images in fMRI studies and relies on regional differences in cerebral blood flow to delineate regional activity. Functional connectivity matrices and hub nodes (central positions of brain information transmission) are typically used for fMRI data analysis. Growing evidence suggests that spontaneous activity of interregional neural synchrony has a coherent structure and may play a role in a host of cognitive and neural processes. Brain functional changes may accompany or even precede detectable structural alterations (Nakamura et al., 2017; Anckaerts et al., 2019), and brain activity and functional connectivity are more consistent with cognitive function than structural imaging and amyloid deposition (Jann et al., 2020; Reas et al., 2020). Thus, exploring the functional changes in ADAD is useful for identifying early biomarkers and potential circuit-based therapeutic targets. fMRI studies have been widely demonstrated in aging (Sala-Llonch et al., 2015; Chen, 2019), late-onset AD (LOAD) (Dickerson and Sperling, 2009; Vemuri et al., 2012), and many other neurodegenerative disorders (Chen, 2019), whereas ADAD-related fMRI studies are relatively insufficient. We will review brain functional changes and potential targets in ADAD based on task-related and resting-state fMRI studies. We systematically searched studies from MEDLINE (The National Library of Medicine), Web of Science, EMBASE, and Cochrane Library inception through 31 December 2021. The characteristics of studies investigating fMRI changes in ADAD are briefly summarized in Table 1.

**TABLE 1:** Characteristics of studies investigating functional magnetic resonance imaging (fMRI) changes in autosomal dominant Alzheimer’s disease (ADAD).

Category	References	Mutated genes involved	Subjects	Ages of mutation carriers (years)	Cognition status of participants	fMRI tasks used/methods of functional connectivity analysis	Mainly affected brain regions	Publication year
**Task-related neural networks**	Mondadori C. R. A. THER et al. (Sala-Llonch et al., 2015)	*PSEN1* C410Y	*N* = 1	20	preclinical stage	face-profession for episodic memory and working memory	enhanced brain activity in left frontal, temporal, and parietal neocortices during learning, retrieval, and novelty detection	2006
	Mondadori C.R.A. et al. (Sala-Llonch et al., 2015)	*PSEN1* C410Y	*N* = 1	45	aMCI	face-profession for episodic memory and working memory	significantly weaker MTL activity as well as many areas of weaker neocortical activity	2006
	Quiroz Y.T. et al. (Chen, 2019)	*PSEN1* E280A	N (mutation carriers) = 20; N (mutation non-carriers) = 19	Mean age ± SD = 33.70 ± 6.01	preclinical stage	face-name paired-associate learning task	Increased activation of the right anterior hippocampus during encoding of novel face-name associations	2010
	Ringman J.M. et al. (Johnson et al., 1998)	*PSEN1* A431E, *PSEN1* L235V, *APP* V717I	N (mutation carriers) = 11; N (mutation non-carriers) = 7	Mean age (range): 29.9 (23 to 43)	preclinical stage	novelty encoding task	decreased BOLD signal in the anterior cingulate gyrus bilaterally and the left frontal pole during a novelty encoding task	2011
	Reiman E.M. et al. (Dickerson and Sperling, 2009)	*PSEN1* E280A	N (mutation carriers) = 20; N (mutation non-carriers) = 24	Mean age ± SD = 22 ± 3	preclinical stage	face-name associative memory encoding and novel viewing and control tasks	significantly greater activation in hippocampal and parahippocampal regions and less deactivation in precuneus and posterior cingulate regions	2012
	Braskie M. N. et al. (Vemuri et al., 2012)	*PSEN1* A431E, *PSEN1* L235V, *APP* V717I	N (mutation carriers) = 18; N (mutation non-carriers) = 8	Mean age (range): 30.9 (19 to 43)	preclinical period to MCI stage	novelty encoding task	no significant difference in fMRI activity between mutation carriers and non-carriers; greater fMRI activity in the fusiform and middle temporal gyri when approaching the familial age of disease diagnosis	2012
	Braskie M. N. et al. (Mondadori et al., 2006)	*PSEN1* A431E, *PSEN1* L235V, *APP* V717I	N (mutation carriers) = 9; N (mutation non-carriers) = 8	Mean age ± SD = 29.8 ± 5.6	preclinical stage	verbal paired associates task	less fMRI activity in the left hippocampus during memory retrieval	2013
	Sala-Llonch R. et al. (Reiman et al., 2012)	*PSEN1* mutations (M139T, K239N, L235R, L282R, L286P, I439S)	N (mutation carriers) = 19; N (mutation non-carriers) = 13	AMC: Mean age ± SD = 39.09 ± 10.74; SMC: Mean age ± SD = 48.91 ± 7.53	preclinical period to dementia stage	visual encoding task	SMC showed reduced activity in regions of the left occipital and left prefrontal cortices, while both AMC and SMC showed increased activity in a region within the precuneus/posterior cingulate	2013
	Quiroz Y.T. et al. (Quiroz et al., 2010)	*PSEN1* E280A	N (mutation carriers) = 18; N (mutation non-carriers) = 19	Mean age ± SD = 13 ± 2	preclinical stage	face-name associative encoding task	less fMRI deactivation of posterior parietal regions during a memory encoding task	2015
**Resting-state networks**	Chhatwal J.F et al. (Cera et al., 2019)	*PSEN1*: 68, *PSEN2*: 5, *APP*: 10	N (mutation carriers) = 83; N (mutation non-carriers) = 37	AMC: Mean age ± SD = 34.64 ± 8.04; early SMC: Mean age ± SD = 44.46 ± 11.74; SMC with dementia: Mean age ± SD = 49.33 ± 9.72	preclinical period to dementia stage	independent component analysis	significantly decreased DMN fcMRI in the precuneus/posterior cingulate and parietal cortices	2013
	Sala-Llonch R. et al. (Reiman et al., 2012)	*PSEN1* mutations (M139T, K239N, L235R, L282R, L286P, I439S)	N (mutation carriers) = 19; N (mutation non-carriers) = 13	AMC: Mean age ± SD = 39.09 ± 10.74; SMC: Mean age ± SD = 48.91 ± 7.53	preclinical period to dementia stage	seed-based correlation analysis	increased frontal connectivity and reduced posterior connectivity in AMC and decreased frontal and increased posterior connectivity in SMC	2013
	Thomas J.B. et al. (Buckner, 2012)	mutations not identified in the text	N (mutation carriers) = 54; N (mutation non- carriers) = 25	CDR = 0: Mean age ± SD = 33.9 ± 8.5; CDR = 0.5: Mean age ± SD = 41.4 ± 10.4; CDR ≥ 1: Mean age ± SD = 49.4 ± 8.7	preclinical period to MCI stage	seed-based correlation analysis	lower functional connectivity in multiple resting-state networks in asymptomatic mutation carriers near anticipated age of symptom onset; largely similar functional connectivity changes owing to advanced AD in LOAD and ADAD	2014
	Quiroz Y.T. et al. (Quiroz et al., 2010)	*PSEN1* E280A	N (mutation carriers) = 18; N (mutation non-carriers) = 19	Mean age ± SD = 13 ± 2	preclinical period	seed-based correlation analysis	no differences in functional connectivity in the whole network metric	2015
	Zhao T. et al. (Acosta-Baena et al., 2011)	*PSEN1*: 16, *PSEN2*: 1, *APP*: 9	N (mutation carriers) = 26; N (mutation non-carriers) = 29	Mean age ± SD = 33.65 ± 4.43	preclinical period	seed-based correlation analysis	decreased connectivity of left precuneus with right precuneus and superior frontal gyrus and decreased connectivity of medial frontal gyrus with middle frontal gyrus	2020
	Quan M. et al. (Moussa et al., 2012)	*PSEN1* mutations (H163R, L282V, L392V, M270L, L271V, M139V, M139L, I213T, A285V, F105I, I100F, K311R, P433S, G111V, L173F, G206S), *PSEN2* (F181I, V214L, M298T, G34S), and *APP* (I716T, V717I, V715M)	N (mutation carriers) = 70; N (mutation non-carriers) = 102	AMC: Mean age ± SD = 36.3 ± 15.7; SMC: Mean age ± SD = 50.5 ± 9.4	preclinical period to dementia stage	seed-based correlation analysis	increased diffusivity of the left hippocampus-PCC circuit in presymptomatic mutation carriers; impaired caudate-rMFG and putamen rMFG circuits in APP gene mutation carriers; increased fiber numbers of putamen-rMFG circuit in PSEN1 gene mutation carriers.	2020
	Ewers M. et al. (Chhatwal et al., 2013)	*PSEN1*, *PSEN2*, *APP*	N (mutation carriers) = 108; N (mutation non-carriers) = 71	Mean age ± SD = 38.0 ± 10.5	preclinical period to dementia stage	seed-based correlation analysis	higher fMRI-assessed system segregation to be associated with an attenuated effect of estimated years from symptom onset on global cognition	2021

*ADAD, autosomal dominant Alzheimer’s disease; PSEN, presenilin; APP, amyloid precursor protein; CDR, clinical dementia rating; aMCI, amnestic mild cognitive impairment; AMC, asymptomatic mutation carrier; SMC, symptomatic mutation carrier; MTL, medial temporal lobe; rMFG, rostral middle frontal gyrus; DMN, default mode network; BOLD, blood oxygen level dependent.*

## Task-Related Neural Networks in Autosomal Dominant Alzheimer’s Disease

Task-related fMRI can be used to study brain activity when a subject is engaged in a given task. Loss of memory, an early and prominent symptom in patients with AD, correlates with parameters of structural or functional brain integrity. Memory tasks are the most widely used in ADAD fMRI studies. The selection of specific neural networks and abnormalities found in fMRI studies depend heavily on the types of memory or behavior tasks engaged. Despite this, the medial temporal lobe (MTL) (Mondadori et al., 2006; Quiroz et al., 2010, 2015; Braskie et al., 2012, 2013; Reiman et al., 2012) has been demonstrated to be selectively impaired in ADAD along with the posterior cingulate cortex (PCC) (Sala-Llonch et al., 2013; Quiroz et al., 2015) which are also regions of early structural and F-18 2-fluoro-2-deoxy-D-glucose positron emission tomography (FDG-PET) biomarkers in ADAD.

A pioneering fMRI study on ADAD related to memory tasks reported only two mutation carriers (*PSEN1* C410Y) (Mondadori et al., 2006). The 20-year-old mutation carrier displayed enhanced brain activity in the left frontal, the temporal, and the parietal neocortices, while activity levels of the 45-year-old mutation carrier were generally low in many left and right hippocampal segments during episodic memory tasks, but not working memory. Increased activity in the middle temporal gyri and the fusiform was also demonstrated during the novelty encoding tasks in other research, as the subjects approached the estimated age of onset (Braskie et al., 2013). The estimated age of onset means that people who carry a familial Alzheimer’s gene tend to develop symptoms at around the same age. This helps researchers predict their disease stage and select participants for clinical trials. Increased activity may be partially explained by neuro hypertrophy, neuroinflammation, and fiber sprouting during the compensatory stage of pathophysiology (Hashimoto and Masliah, 2004; Stern et al., 2004; Sperling et al., 2010; Zott et al., 2018). These studies suggest that increased fMRI activity in the temporal lobe may be related to incipient ADAD processes, and episodic memory tasks may potentially be manageable to provoke brain activity in the pre-clinical phase of AD (the stage occurring before a clinical diagnosis of a cognitive disorder). The studies have also shown decreased activity of the temporal region in cognitively unimpaired ADAD mutation carriers. In the verbal paired-associate task fMRI study, pre-symptomatic mutation carriers showed less fMRI activity in the hippocampus and the posterior middle temporal gyrus than controls during retrieval (Braskie et al., 2013). This may be due to the different tasks or the older age of the participants in the latter study. Overall, the hippocampus, the middle temporal gyri, the fusiform, and the middle temporal gyri presented early activity changes in fMRI in subjects at risk for ADAD. It has been reported that the hippocampus can interact with the fusiform gyrus to participate in cognitive networks. Activation in the hippocampus may be related to the novelty of the stimulus, while the fusiform gyrus has been proven to be important in novelty encoding (Yamaguchi et al., 2004), object processing (Tyler et al., 2004), and recognition of complex visual stimuli (Stern et al., 1996).

As for the PCC and parietal region, asymptomatic mutation carriers showed increased BOLD activity in the posterior cingulate and symptomatic mutations carriers showed increased activity in the lingual gyrus, with precuneus cortex activity increased in both the asymptomatic and symptomatic mutation carriers during the visual memory encoding task, compared to non-mutation carriers (Sala-Llonch et al., 2013). In a verbal paired-associate task-related fMRI study, less signal in the inferior parietal cortex and the precuneus during retrieval, not encoding, was found in pre-symptomatic mutation carriers. Even after controlling for gray matter volume as a covariate, there were still significant differences in these regions, which indicated that atrophy could not explain the entire effect (Braskie et al., 2013). The PCC, precuneus, and parietal cortex are involved in many cognitive functions, including episodic memory, working memory, the focus of attention, visuospatial processing, etc. The involvement of the precuneus/PCC is significant in the development of ADAD. Early amyloid deposition, abnormal structure, and reduced metabolism in these regions have been observed in ADAD (Johnson et al., 1998; Quiroz et al., 2018; Yokoi et al., 2018; Sanchez et al., 2021). Changes in the PCC and parietal cortex in task-related fMRI in asymptomatic subjects at risk for ADAD further suggest that the posterior cortical regions play key roles in the development of dementia.

The anterior cingulate gyrus and frontal lobes are also vulnerable regions in the functional imaging studies of ADAD. During the novelty encoding trials (Ringman et al., 2011), decreased BOLD signals in the anterior cingulate gyrus bilaterally and the left frontal pole were found in pre-symptomatic mutation carriers, compared with matching cognitively normal family members. In addition, decreased BOLD activity in regions of the left middle and inferior frontal gyri, the left frontal operculum, and the left lateral occipital cortex was also found in symptomatic mutation carriers during the visual memory encoding task (Sala-Llonch et al., 2013). The anterior cingulate correlates with the striatum *via* frontal-subcortical circuits (Ringman et al., 2011), and abnormal frontostriatal connectivity in both *APP* and *PSEN1* mutation carriers and its association with general cognitive function were also reported by Quan et al. using rs-fMRI. The anterior cingulate and frontal regions are involved in certain higher-level functions, such as attention allocation, decision-making, emotion, and memory. The anterior cingulate is also part of the salience network (Seeley et al., 2007), which might be selectively activated during the processing of novel stimuli. Decreased glucose metabolism and cerebral blood flow in the anterior cingulate in pre-symptomatic subjects carrying *PSEN1* mutations (Johnson et al., 2001), abnormalities in functional connectivity in aging, and mild cognitive impairment have been described (Cera et al., 2019).

A series of task-related fMRI studies were conducted in the big Colombian pedigree (*PSEN1* E280A) to explore brain activity in children (9–17 years old) (Quiroz et al., 2015), young adults (18–26 years old) (Reiman et al., 2012), and middle-aged adults (average age 33.7 years old) (Quiroz et al., 2010), who were all younger than 44 years old (mean age at onset of the disease (Acosta-Baena et al., 2011)). The encoding and viewing of novel face-name pairs (associative memory functioning task) were employed among these subjects. The analysis focused on the hippocampal system in the middle-aged adult group. Compared to non-carriers, cognitively intact *PSEN1* mutation-carrying adults demonstrated hyperactivation within the right anterior hippocampus during the encoding of novel associations. In young adults, mutation carriers showed significantly greater activation in the hippocampal and parahippocampal regions and less deactivation in the precuneus and posterior cingulate regions. Interactions remained significant in the right hippocampus, the right parahippocampal gyrus, the right precuneus, and the right posterior cingulate regions, after correction for multiple comparisons. Compared to non-carriers, mutation-carrying children appeared to have less deactivation in the posterior parietal regions. However, there were no significant differences between the groups in MTL activation, either when controlling for age or when looking at age-related slopes. In summary, compared to non-carriers, *PSEN1* E280A mutation carriers showed less deactivation in the precuneus and posterior cingulate regions during teenager and young adulthood, while the increased activity of the hippocampal and parahippocampal regions began from early adulthood and lasted until middle adulthood during memory association tasks.

In general, abnormalities in brain activity were found in the hippocampus, the MTL, the posterior cortex, the anterior and posterior cingulate cortices, and the middle and inferior frontal regions. These regions are also critical for cognitive function and show structural and pathological changes during the early phase of ADAD. The results of different studies vary in the affected brain regions and changes in activity. Possible explanations for these inconsistent results may be due to discrepant tasks, different populations, subjects’ ages, and levels of cognition. Unified and standard task performance is needed in future studies when conducting meta-analyses.

## Resting-State Networks in Autosomal Dominant Alzheimer’s Disease

Resting-state functional magnetic resonance imaging, which evaluates brain regional interactions during the resting condition, can be used to study patterns of intrinsic brain connectivity or functional work *in vivo*. rs-fMRI measures spontaneous low-frequency fluctuations in the BOLD signal to investigate the functional architecture of the brain and allows the identification of various resting-state networks, or spatially distinct areas of the brain that demonstrate synchronous BOLD fluctuations at rest. Neural networks in rs-fMRI include several components (Moussa et al., 2012), with the default mode network (DMN) being the most studied and easily visualized network (Buckner, 2012). The DMN involves the precuneus and PCC, the parietal and temporal cortices bilaterally, the medial prefrontal cortex (mPFC), and some regions within the hippocampal memory system. Exploring the changes in restating state networks in ADAD may provide non-invasive functional imaging biomarkers for early diagnosis and interventions in ADAD.

In a DMN study of children, who were much younger than the estimated age of onset, in the big Colombian pedigree (Quiroz et al., 2015), no differences in functional connectivity in the whole network metric (six pre-defined nodes) were found. However, greater functional connectivity between the PCC and the bilateral MTL regions was found in mutation carriers than in non-carriers. No reduction in functional connectivity was observed in the mutation carriers. The opposite results were found in other studies using the independent component correlation algorithm (Chhatwal et al., 2013), in which functional connectivity within much of the DMN was decreased in ADAD mutation carriers compared with non-carriers. The most apparent decreased connectivity was with the major posterior node of the DMN (precuneus/posterior cingulate, PPC), along with the anterior node of the DMN (mPFC) and bilateral parietal cortices. Furthermore, compared with non-mutation carriers, functional connectivity in the PPC and right parietal cortex was decreased in asymptomatic mutation carriers, whereas decreased functional connectivity in the PPC, mPFC, and the left and right parietal cortices was observed in symptomatic mutation carriers. More complex and divergent results were reported by Sala-Llonch et al. (2013). The anterior components of the DMN were increased in asymptomatic subjects and decreased in symptomatic subjects, while the posterior components of the DMN were decreased in asymptomatic subjects and increased in symptomatic subjects, compared with controls. However, these studies also found that decreased functional connectivity within both the posterior and anterior DMN in the pre-symptomatic stage of ADAD, and connectivity disruption within the posterior DMN was disrupted earlier (Zhao et al., 2020). Quan et al. (2020) provided novel evidence of changes in neural circuits in ADAD using rs-fMRI, combined with T1 structural MRI and diffusion tensor imaging. The anterior resting networks and frontostriatal circuits were affected in the asymptomatic stage of ADAD. More narrowly, frontostriatal circuits were impaired and functional connectivity was decreased in *APP* mutation carriers, while the connectivity of the putamen-rostral middle frontal gyrus (putamen-rMFG) showed increases in *PSEN1* mutation carriers. In summary, both anterior and posterior functional connectivity of the DMN can be affected in pre-symptomatic and symptomatic subjects, with the PCC, MTL regions, and pre-frontal cortex being more prominent. Meanwhile, altered functional connectivity in mutation carriers has been observed for several years or even over a decade before symptom onset (Sala-Llonch et al., 2013; Thomas et al., 2014). Furthermore, a significant interaction between mutation carrier status and estimated years from symptom onset (EYO) with a significant negative correlation between fMRI and EYO in mutation carriers was also demonstrated, but not in non-carriers. Interestingly, less fMRI deactivation of the parietal regions in the absence of MTL alterations was also observed, which suggests that functional abnormalities in the parietal region may precede MTL changes early in the disease. A hypothesis has been raised that higher segregation of the functional networks represents a functional mechanism underlying cognitive resilience in AD, and higher fMRI-assessed system segregation was found to be associated with an attenuated effect of EYO on global cognition (Ewers et al., 2021).

Differences in resting-state networks were compared between ADAD and aging, and between ADAD and LOAD. In general, the degradation pattern of functional connectivity in ADAD was similar to that in LOAD (Thomas et al., 2014), but it was different from that in aging (Chhatwal et al., 2018). Inter- and intra-network functional connectivity decreases with increasing Clinical Dementia Rating (CDR) were similar for both ADAD and LOAD in multiple resting-state networks, including DMN. With some subtle differences observed, there was a modestly greater effect on disease severity seen in ADAD than in LOAD, which possibly reflected a faster spread of pathology across diseased connections in ADAD. As for ADAD versus aging, cognitive networks were preferentially degraded in ADAD, with changes in motor and visual networks seen only in the advanced stages. A contrasting degradation pattern was observed in aging subjects, where visual networks degraded to a similar or greater degree than cognitive networks. Both studies also demonstrated that inter-network connections were preferentially targeted in ADAD, with intra-network connections relatively spared. The results suggest that particular networks are selectively vulnerable in ADAD and support the ‘network diffusion’ models of AD progression (Raj et al., 2015), which preferentially degrades connections within cognitive networks. This evidence supports that aging and AD demonstrate distinct pathophysiological mechanisms and that ADAD may serve as an effective model to study LOAD pathophysiology.

## Conclusion and Perspectives

The hippocampus, the medial temporal region, the precuneus, the inferior parietal cortex, and the anterior and posterior cingulate cortices are key hubs in both task-related and rs-fMRI in ADAD. Inter- and intra-networks in these regions, along with the striatum, present abnormal functional connectivity in the early pre-symptomatic stages of ADAD, with the former being much more prominent. The regional brain activity and functional connectivity decrease as subjects carrying mutations approach the estimated age of onset or cognitive function gradually deteriorates.

As for neural reserves, most previous studies have been conducted in aging and SAD populations, while similar studies in ADAD are scarce. A recent study demonstrated that higher levels of global functional connectivity of the left frontal cortex were associated with attenuating effects of AD pathology on cognition in prodromal ADAD (mild cognitive impairment due to AD plus very mild AD dementia) (Franzmeier et al., 2018). It has been demonstrated that increased connectivity of frontal hubs is associated with cognitive training (Takeuchi et al., 2017), transcranial magnetic stimulation, transcranial direct-current stimulation (Chen et al., 2013; Kim et al., 2016), or physical exercise (Duzel et al., 2016), and increased hub connectivity is associated with better cognition (Cole et al., 2012; Liu et al., 2017). ADAD provides a good vehicle for conducting early and longitudinal neuroimaging studies or intervention studies to further elucidate how neural reserve and compensation evolve at different stages of ADAD and the relationship between cognitive reserve and its neural correlates. Exploring the mechanism under the neural network-level treatment helps to discover the potential molecular targets of ADAD, which helps in the development of drug therapy.

Considering that ADAD accounts for less than 1% of all AD cases, fMRI studies on ADAD are insufficient and the results are inconsistent. These findings are difficult to integrate due to different choices of subjects, tasks, regions of interest, etc. Moreover, cross-center effects are not yet widely recognized. ADAD is a subtype of rapidly progressive dementia with certain heterogeneity, and its fMRI characteristics require more detailed hierarchical analysis based on age, genotype, biomarkers, etc. is warranted. Future fMRI studies in ADAD are needed to optimize more specific connectivity composites, investigate the network-level neural underpinnings of cognitive alterations, and clarify the chronological order of fMRI changes and other pathophysiological biomarkers of ADAD progression. Using multi-network or inter-network composite connectivity measures may be particularly useful for increasing the specificity of functional connectivity MRI biomarkers and reducing the impact of common confounding conditions. Moreover, technical factors are critical in assessing fMRI: small amounts of head movement that are not easily detected and multiple different methods of data analysis can confound the results. Subsequent studies can compare different techniques to obtain sensitive and stable parameters and combinations of parameters and sites for detecting microstructural changes. Reliable multi-center studies will bring major progress in fMRI research on ADAD.

## Author Contributions

The author confirms being the sole contributor of this work and has approved it for publication.

## Conflict of Interest

The author declares that the research was conducted in the absence of any commercial or financial relationships that could be construed as a potential conflict of interest.

## Publisher’s Note

All claims expressed in this article are solely those of the authors and do not necessarily represent those of their affiliated organizations, or those of the publisher, the editors and the reviewers. Any product that may be evaluated in this article, or claim that may be made by its manufacturer, is not guaranteed or endorsed by the publisher.
